# Folding of the Ig-Like Domain of the Dengue Virus Envelope Protein Analyzed by High-Hydrostatic-Pressure NMR at a Residue-Level Resolution

**DOI:** 10.3390/biom9080309

**Published:** 2019-07-26

**Authors:** Tomonori Saotome, Maxime Doret, Manjiri Kulkarni, Yin-Shan Yang, Philippe Barthe, Yutaka Kuroda, Christian Roumestand

**Affiliations:** 1 Department of Biotechnology and Life Science, Tokyo University of Agriculture and Technology, 2-24-16, Nakamachi, Koganei, Tokyo 184-8588, Japan; 2 Centre de Biochimie Structurale, CNRS UMR 5048, University of Montpellier-INSERM U 1054, 29 Rue de Navacelles, 34090 Montpellier, France

**Keywords:** high-hydrostatic-pressure nuclear magnetic resonance, thermodynamic stability, protein folding

## Abstract

Dengue fever is a mosquito-borne endemic disease in tropical and subtropical regions, causing a significant public health problem in Southeast Asia. Domain III (ED3) of the viral envelope protein contains the two dominant putative epitopes and part of the heparin sulfate receptor binding region that drives the dengue virus (DENV)’s fusion with the host cell. Here, we used high-hydrostatic-pressure nuclear magnetic resonance (HHP-NMR) to obtain residue-specific information on the folding process of domain III from serotype 4 dengue virus (DEN4-ED3), which adopts the classical three-dimensional (3D) ß-sandwich structure known as the Ig-like fold. Interestingly, the folding pathway of DEN4-ED3 shares similarities with that of the Titin I27 module, which also adopts an Ig-like fold, but is functionally unrelated to ED3. For both proteins, the unfolding process starts by the disruption of the N- and C-terminal strands on one edge of the ß-sandwich, yielding a folding intermediate stable over a substantial pressure range (from 600 to 1000 bar). In contrast to this similarity, pressure-jump kinetics indicated that the folding transition state is considerably more hydrated in DEN4-ED3 than in Titin I27.

## 1. Introduction

Dengue fever, endemic in tropical and subtropical regions, is a significant public health problem in Southeast Asia [1], and no effective vaccine or specific medication is available [2]. Over 33 million cases of dengue fever with several death cases are reported every year [3]. Dengue fever is caused by the mosquito-borne dengue virus (DENV), which is an enveloped, single-stranded, positive-sense RNA flavivirus. Four distinct, but closely related, dengue serotypes have been identified. Infection by a single serotype provokes a mild to high fever for a few days, after which the patient usually recovers steadily and gains a long-lasting immunity against the infecting serotype. However, subsequent heterotypic infections increase the risk of developing dengue haemorrhagic fever (DHF) and shock syndrome (DHS) with a ~5% mortality rate and severe complications [4,5]. 

DENV’s genome comprises ten genes encoding for the capsid (C), premembrane (prM), envelope (E), and seven nonstructural (NS) proteins [6]. The envelope is formed by the association of 180 E-protein units and constitutes the outer surface of the dengue virus particle. The E-protein mediates virus–host attachment and also contains the binding site for neutralizing antibodies [7,8]. It is composed of three domains: (ED1–3) [9].

The ED3 domain of E-protein is a ∼100-residue globular ß-sheet protein that adopts the fold of an Ig-like domain. It contains two putative dominant epitopes recognized by antibodies, and a region that can affect the binding to the heparin sulfate receptor, which drives the virus entry into the host cell [9,10]. As such, ED3 plays a critical role in viral infection. Phylogenetic studies show that DEN4 is the oldest of the four DEN viruses [11]. Surprisingly, only eight amino acid mutations in ED3 separate the two most divergent DEN4 strains, among which five have been implicated in the emergence of the human DEN4 virus [11,12] (Supplementary Materials, Figure S1 and Table S1). Recent X-ray crystallography (DEN3-ED3, DEN4-ED3) [13,14] and nuclear magnetic resonance (NMR) (DEN4-ED3) [15] analysis demonstrated that dengue ED3 remains natively folded even when it is isolated from the rest of the E-protein. Moreover, previous studies indicated that residues in the hydrophobic core of ED3 were not fully conserved among the four serotypes [13] and that a rearrangement of the side-chains interdigitization ensured the stabilization of the protein core and thus of the natively folded structure of DEN-ED3 across serotypes [14]. More generally, the Ig-fold is a classical fold found in several proteins with unrelated functions, and it would be of interest to determine whether proteins with Ig-fold but different core packing exhibit similar folding properties at the residue level [16].

The combination of high hydrostatic pressure with NMR has proven to be a powerful tool to obtain high-resolution structural information about protein folding [17,18,19,20,21,22,23,24,25]. Indeed, multidimensional NMR intrinsically provides multiple residue-specific probes, while the reversibility of high-pressure folding/unfolding experiments ensures the proper characterization of thermodynamic parameters. In addition, contrary to chemical denaturants or temperature, pressure does not affect the protein uniformly: it acts by reducing volume, primarily by eliminating solvent-excluded voids. Since these voids are heterogeneously distributed throughout the protein 3D structure, certain regions are more pressure-dependent than others, thus favoring the characterization of folding intermediates [17,26].

In the present study, we used high-pressure NMR spectroscopy to monitor the unfolding of ED3 from dengue-4 virus (DEN4-ED3) and shed insight into the structural details of its folding pathway. In particular, we found that the folding pathway of DEN4-ED3 shares similarities with that of the I27 module from the giant multimodular protein Titin found in the striated muscle cells [27], which also adopts an Ig-like fold. In both proteins, unfolding starts with the disruption of the N- and C-terminal strands on the edge of the ß-sandwich, but the Transition State Ensemble (TSE) of DEN4-ED3 was found to be significantly more hydrated, and hence less structured, than that of Titin I27.

## 2. Results 

### 2.1. Setting the Physical–Chemical Conditions for ED3 Unfolding

The DEN4-ED3 domain did not completely unfold in the NMR buffer, at pH 7, in a 5–40 °C temperature range, and in the pressure range allowed by the experimental set-up (1–2500 bar). Thus, we decided to destabilize the protein slightly by adding a subdenaturing concentration of a chaotropic reagent (guanidinium chloride). 

**Figure 1:**
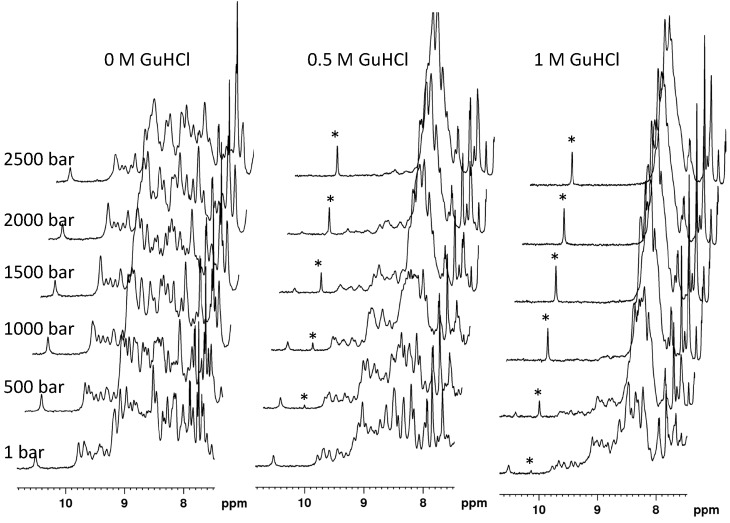
Determination of the experimental conditions (guanidine concentration) for the denaturation study of DEN4-ED3. Stacked plot of 1D ^1^H spectra (amide region) recorded at variable pressure (from bottom to top) and variable concentration (0, 0.5, and 1 M, from left to right) of guanidine. Protein unfolding was monitored by following the rise of the indolic resonance of Trp103 in the unfolded state (indicated with an asterisk) and the concomitant decrease of the same resonance of Trp103 in the folded state (left: most resonance).

The indolic proton HN resonances of the native and unfolded protein species were used to monitor DEN4-ED3 unfolding with pressure, using 1D NMR spectroscopy (Figure 1): they are well individualized and do not overlap with HN amide resonances in the 1D spectrum of DEN4-ED3, and thus easy to follow. After different trial-and-error assays, adjusting the concentration of GuHCl and the temperature, we found that the protein completely unfolds in the 1–2500 bar pressure range at pH 7 (Tris 10 mM), 10 °C, and at a concentration of GuHCl of 0.5 M (Figure 1).

Note that the concomitant decrease of the folded-state indole HN resonance of Trp103 and the increase of the corresponding resonance in the unfolded state strongly support a slow exchange between the chemical environments this residue experiences in the folded and unfolded states: the fit of the pressure dependence of these two resonances with a two-state equilibrium (Equation (1)) yields ∆*V_f_*^0^ and ∆G^0^ values within experimental uncertainty.

### 2.2. Assignment of the Amide Protons

**Figure 2:**
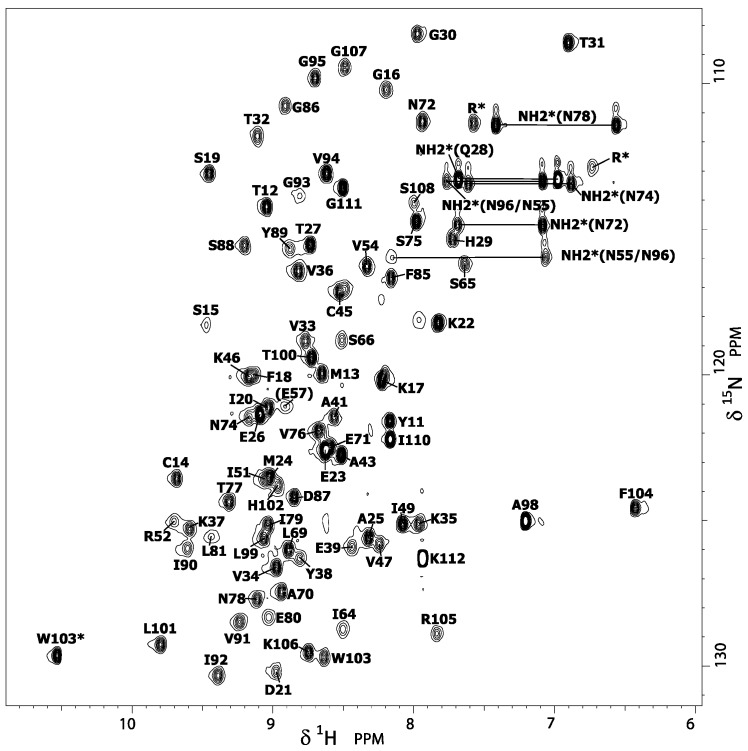
DEN4-ED3 nuclear magnetic resonance (NMR) fingerprint. {^1^H-^15^N} HSQC spectrum (see Materials and Methods section) of DEN4-ED3 at 600 MHz, 10 °C, pH 7.0, recorded on a 1 mM, ^15^N-uniformly labeled sample dissolved in a 10 mM Tris buffer (0.5 M guanidine). Cross-peak assignments were indicated using the one-letter amino acid and number code.

**Figure 3:**
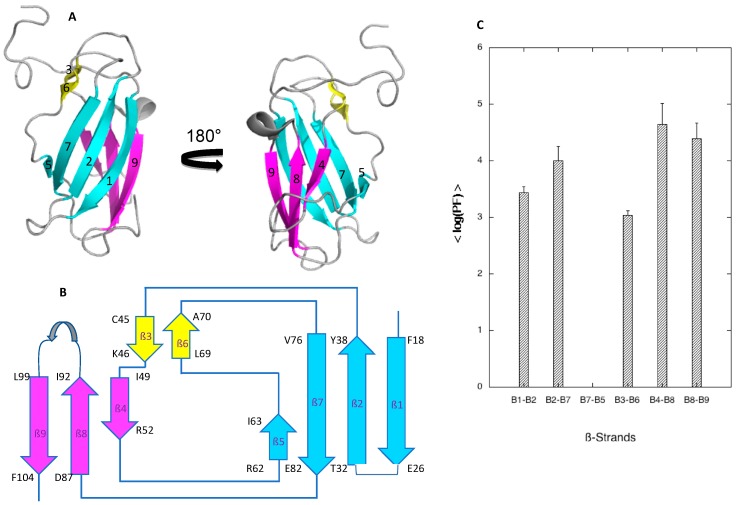
Structure and local stability of DEN4-ED3. (**A**) Ribbon representation of the solution structure of DEN4 (Volk et al. [15]). The cyan and magenta colors have been used for the two ß-sheets that form the ß-sandwich. Note the presence of an extra small double-stranded ß-sheet that does not contribute to the structure of the ß-sandwich. (**B**) Topology diagram of DEN4-ED3. The same colors as in (**A**) have been used for the different ß-sheets. (**C**) Average protection factor (<PF>) values calculated for amide protection factors stabilizing each ß-sheet in the ß-sandwich.

The solution structure of DEN4-ED3 was solved previously by NMR (Protein Data Bank (PDB) entry: 2h0p) using a complete set of {^1^H,^13^C,^15^N} triple-resonance experiments for chemical-shifts assignment (deposited at BRMB: 7087) [15]. This structure was determined at 25 °C and pH 7.5 (50 mM Tris buffer, 50 mM NaCl), under physical and chemical conditions slightly different than those used in the present denaturation study (pH 7, 10 °C, no salt, and 0.5 M GuHCl), justifying the reassignment of the amide group resonances. Thus, from ^15^N-Edited nuclear Overhauser effect spectroscopy (NOESY) and total correlation spectroscopy (TOCSY) three-dimensional (3D) experiments, the classical sequential assignment strategy was used to reassign the amide proton and nitrogen resonances (and, besides, the Hα resonances) displayed in the {^1^H,^15^N} HSQC spectra further used for the denaturation study (Figure 2). These minimal changes in chemical and physical conditions result in very slight differences in chemical shifts (Supplementary Materials, Figure S2), suggesting that the Ig-like 3D structure of DEN4-ED3 (Figure 3A) is not altered by the presence of guanidinium chloride. Thus, the 3D structure previously determined by Volk et al. [15] was used as a template for displaying the results obtained in the present study.

### 2.3. Local Stability Probed by Proton/Deuteron Exchange Measurements.

Local stability is important information when studying protein unfolding, since it is often related to partial unfolding of the protein. H/D exchange measurements can provide such information since they give access to information on the strength of the H-bonds, hence on the stability of the secondary structure elements. H/D exchange for DEN4-ED3 was investigated in our experimental conditions (pH 7 and T = 10 °C), but in the absence of chemical denaturant, to explore the local stability of the protein. To this aim, {^1^H,^15^N} HSQC spectra were recorded with time on a protein sample freshly dissolved in D_2_O (see Materials and Methods for details). After protein dissolution, 25 amide cross-peaks were still apparent in the first HSQC recorded, but accurate fit with an exponential decay can be obtained for the HSQC cross-peak intensity of only 22 residues (18 involved in the H-bonds stabilizing the ß-sheets) over 107 nonproline residues. Their corresponding protecting factors (PFs) were calculated from the exchange rate constants obtained from the fit [28] (Supplementary Materials, Table S2). The local stability of the different ß-sheets was then estimated by the average value of the protecting factors (<PF>) calculated over the amide protons involved in the main-chain H-bond stabilizing the ß-sheet (Figure 3). 

These results reveal that the ß4-ß8 and ß8-ß9 sheets, which form a triple-stranded ß-sheet on one face of the ß-sandwich, appear significantly more stable (4.4 < average log(PF) < 4.6) than the ß1-ß2 and ß2-ß7 sheets on the other face of the ß-sandwich (3.4 < average log(PF) < 4). Although amide cross-peaks of residues involved in the main-chain stabilizing H-bonds were present in the first HSQC spectrum recorded on the freshly dissolved protein sample, the corresponding PF cannot be measured for the short ß5-ß7 sheet (the ß5 strand consists only of two residues: Arg62-Ile63) due to a rapid decrease of the cross-peak intensities with time. This means that this sheet is extremely unstable and probably only transiently formed. Note that the ß3-ß6 sheet, also extremely short since it involves only two residues in each strand (Cys45-Lys46 on ß3 strand, Leu69-Ala70 on ß6 strand), shows a stability only slightly weaker than observed for the ß-sandwich (aver. log(PF) ≈ 3), even if it does not contribute to its structure by itself.

### 2.4. High-Pressure Unfolding Monitored with Nuclear Magnetic Resonance Spectroscopy: Steady-State Studies

**Figure 4:**
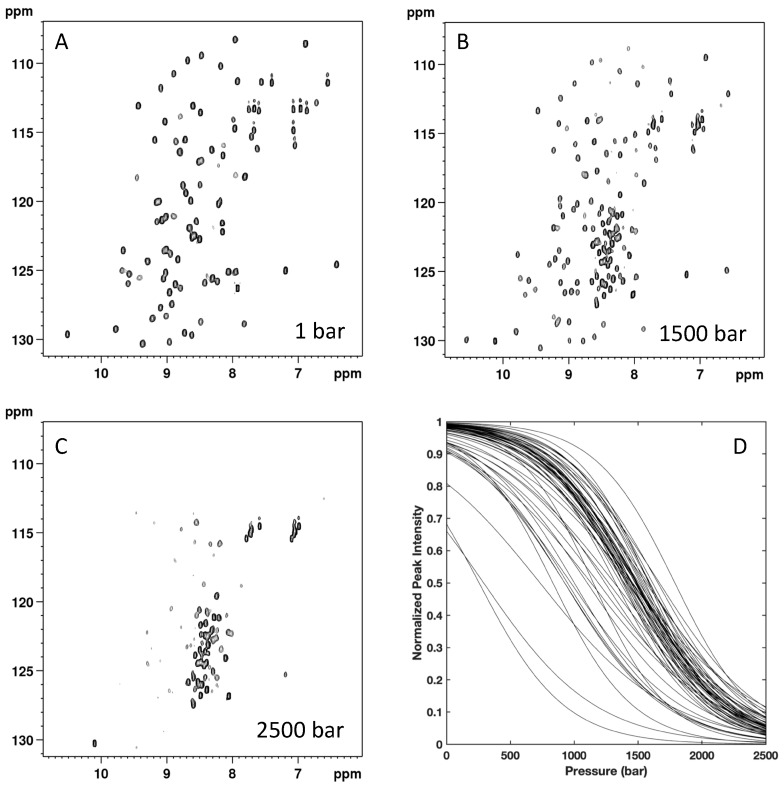
Nuclear magnetic resonance-detected high-pressure unfolding of DEN4-ED3 at 10 °C and 0.5 M guanidinium chloride. (**A–C**) Examples of {^1^H-^15^N} HSQC NMR spectra at different pressures as indicated; (**D**) overlay of the normalized residue-specific denaturation curves as obtained from the fit of the pressure-dependent sigmoidal decrease of the residue cross-peak intensities in the HSQC spectra with Equation (1).

**Figure 5:**
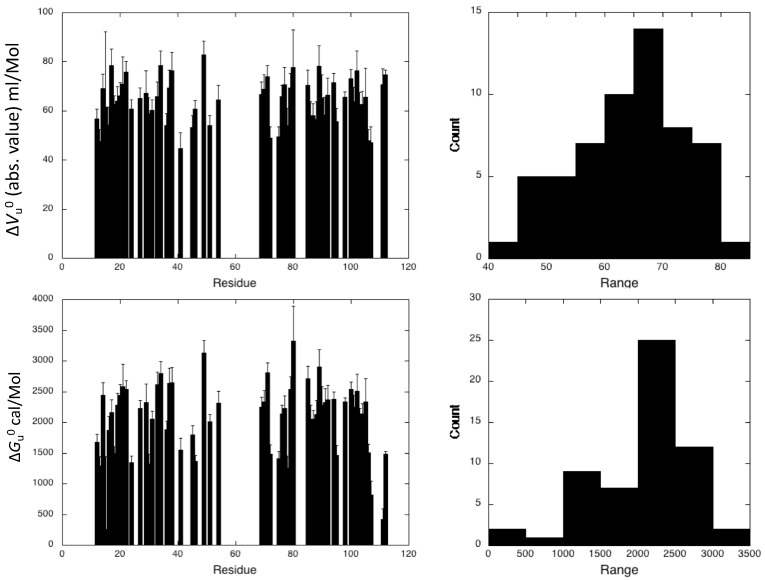
Steady-state thermodynamic parameters. (Right) Apparent residue-specific ∆*V_u_*^0^ (absolute values) (top) and ∆*G_u_*^0^ (bottom) values obtained through the fit of the intensity decrease of the 2D {^1^H-^15^N} HSQC cross-peaks with pressure recorded on DEN4-ED3, plotted versus the protein sequence. (Left) Distribution of the values of ∆*V_u_*^0^ (top) and ∆*G_u_*^0^.

To further probe the structural details of DEN4-ED3 folding, two-dimensional (2D) {^1^H,^15^N} HSQC spectra of ^15^N uniformly labeled DEN4-ED3 in the presence of 0.5 M guanidinium chloride were recorded as a function of pressure (Figure 4). As typically observed, the overall intensity of each native state peaks decreased as a function of pressure, while the intensity of the corresponding unfolded state peaks, centered around 8.5 ppm in the proton dimension, concomitantly increased. This strongly suggests a slow equilibrium between the chemical environment of each residue in the native and unfolded state on the NMR timescale, as well as a two-state transition between each native/unfolded pairwise cross-peak during the unfolding process. Thus, locally, the loss of intensity for each native state cross-peak can be interpreted as a two-state transition, even though the global protein unfolding does not conform to such a model [17]. Since resonance assignments were available only for the native state structure of DEN4-ED3, the decrease in intensity with pressure for each corresponding cross-peak was fitted to a two-state, pressure-induced unfolding model as described in the Materials and Methods (Equation (1), Figure 4), yielding residue-specific values for the apparent volume change (∆*V*_f_^0^) and apparent free energy (∆*G*^0^) of unfolding [17] (Figure 5). Accurate fitting (Supplementary Material, Figure S3) was obtained for 58 residues of the 107 nonproline residues, providing a substantial number of local probes for the description of the DEN4-ED3 folding pathway. The fitted curves correspond to residues for which the cross-peaks do not overlap either in the folded state of the protein or in between the folded and unfolded states. Residues that exhibit cross-peaks with very low intensity at atmospheric pressure, probably due to slow or intermediate exchange between different populations, cannot be fitted accurately and were discarded from the analysis.

The DEN4-ED3 exhibits moderate stability at 0.5 M guanidinium chloride, with an average value <∆*G_u_*^0^> = 2.23 ± 0.08 kcal/mol. Besides, an average <∆*V_u_*^0^> value of −64 ± 9 mL/mol was obtained for the construct. While the two-state model was adequate for all individual residue-specific unfolding curves, different profiles were observed for different residues, demonstrating significant deviation from two-state behavior of the global unfolding for the protein. The asymmetric distributions of apparent ∆*V_u_*^0^ and apparent ∆*G_u_*^0^ (Figure 5) are indicative of partial unfolding of the ED3 domain at intermediate pressures. 

Fractional contact maps were constructed for DEN4-ED3 to visualize which regions of the protein become disordered at intermediate pressures [17]. The probability of contact for any pair of residues, *P_i,j_*, at a given pressure was defined as the geometric mean of the fractional intensity of the HN resonances of each of the two residues in the folded state at the same pressure (*P_ij_* = (*P_i_* × *P_j_*)^1/2^), extracted from the normalized residue-specific denaturation curves [21]. 

**Figure 6:**
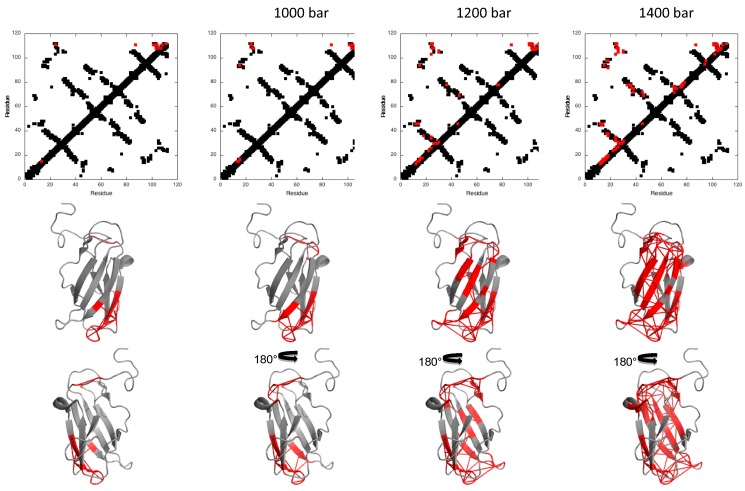
Pressure denaturation of DEN4-ED3. (Top) Contact maps built from the solution structure obtained DEN4-ED3 [15] at 600, 1000, 1200, and 1400 bar, as indicated. The contacts above the diagonal have been colored in red when contact probabilities (*Pij*) lower than 0.5 are observed. (Bottom) Ribbon representations of the solution structure DEN4-ED3 where the red ticks represent contacts that are weakened (*Pij* ≤ 0.5) at the corresponding pressures.

The pressure dependence of the contact maps shows that the first region affected by an increase of pressure implicates one edge of the ß-sandwich, namely the junction made by the ß-strands 1 and 9, corresponding to the N- and C-termini of the molecule. A significant (*P_ij_* ≤ 50%) loss of contacts is already apparent at 600 bar (Figure 6), and up to 1000 bar, this partial unfolding concerns mainly this region. Above this pressure, partial unfolding extends throughout the ß-sandwich, probably as a result of water penetration in the hydrophobic core of the protein. Interestingly, unfolding propagates first on one face of the ß-sandwich (strands 1, 2, 7), while the other face (strands 4, 8, 9) remains almost unaffected until 1400 bar. Note that, from proton/deuteron exchange experiments, the ß1-ß2-ß7 sheet is less stable than the ß4-ß8-ß9 sheet; thus, partial unfolding seems to map to the zone of fragility in the ß-sandwich. 

### 2.5. High-Pressure Unfolding Monitored with Nuclear Magnetic Resonance Spectroscopy: Kinetics Studies

To further probe the folding reaction, we performed a kinetics study by recording series of 2D {^1^H,^15^N} SOFAST-HMQC experiments (2 min measuring time each) [29] over 2 h after 200 bar positive pressure jumps. From the exponential decrease in intensity with time of each native state cross-peaks in these real-time 2D NMR experiments, it is in principle possible to extract the folding/unfolding residue-specific relaxation time τ. This measurement can be repeated after similar P-jumps between different pressures, and the different values of τ obtained further plotted versus pressure, yielding the so-called residue-specific “chevron plots” that allow extracting residue-specific rate constants of folding *k*_f_ and unfolding *k*_u_, as well as residue-specific activation volumes of folding (∆*V*_f_^‡^) and unfolding (∆*V*_u_^‡^) [24]. Among these results, the residue-specific values of ∆*V*_f_^‡^ are of special importance, since they provide an interesting description of the hydration state of the transition state ensemble (TSE), and more precisely, which residue is hydrated (volumetrically close to the unfolded state) or dehydrated (volumetrically close to the folded state) at the TSE. Unfortunately, in the case of DEN4-ED3, the weak exponential decrease in intensity with time observed for most of the native state cross-peaks after P-jumps in the real-time 2D NMR experiments precluded an accurate determination of the residue-specific relaxation times *τ*, and, consequently, an accurate determination of the residue-specific values of ∆*V*_f_^‡^.

**Figure 7:**
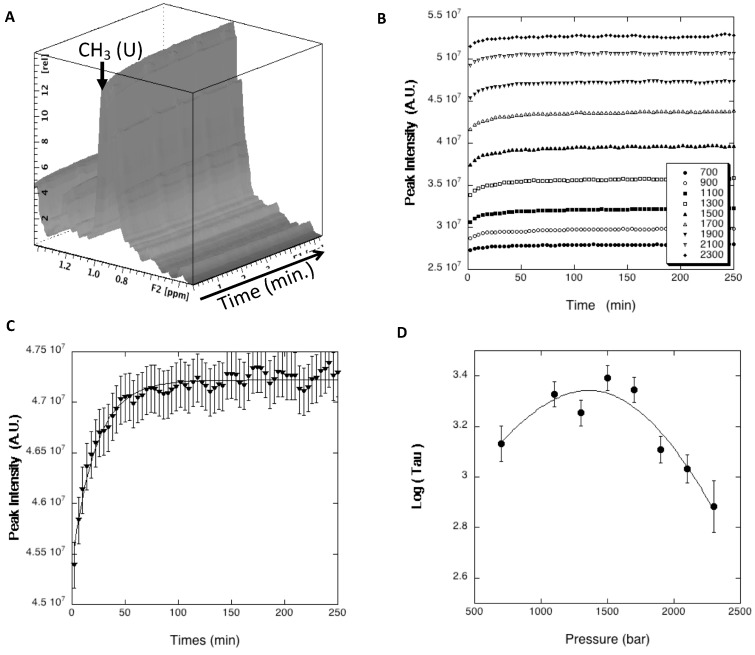
P-jump experiments followed by high-pressure 1D proton NMR. (**A**) Stacked plot of the 1D proton NMR spectra recorded on DEN4-ED3 after a positive 200 bar P-jump (1700 to 1900 bar). Only the region of the methyl resonances is reported. The arrow indicates the resonance corresponding to unfolded methyl groups (CH3 (U)), used as a probe to measure the relaxation rates *τ*_(*p*)_. (**B**) Time evolution of the unfolded methyl resonance (CH3 (U)) after positive 200 bar P-jumps realized between different pressures (final pressures are reported in the insert). (**C**) Example of fit obtained for the 1700 to 1900 P-jump: the data points were fitted with an exponential growth to obtain the values of *τ*. (**D**) The fit with Equation (3) of the chevron plot of log(*τ*) versus pressure allows extraction of the folding and unfolding global rate constants *k*_u_ and *k*_f_, as well as the apparent global residue-specific activation volume of folding (∆*V*_f_^‡^) (see Material and Methods).

Nevertheless, the NMR measurement of global folding kinetic parameters remains possible. For this, we recorded series of 1D proton NMR spectra after P-jumps [25,27]. The proton resonance at 0.96 ppm, corresponding to unfolded-state methyl proton resonances, was used as a probe to measure the global relaxation times τ (Figure 7A). Indeed, it provides a much more sensitive probe to monitor the folding/unfolding reaction than the amide cross-peaks that correspond each to a single proton, but at the expense of the sequence specificity. Thus, global values of *τ* can be accurately determined from the exponential growth of this resonance after P-jumps (Figure 7B,C), yielding reliable global values for ∆*V*_f_^‡^, ∆*V*_u_^‡^, *k*_u_, and *k*_f_.

A value of ∆*V*_f_^‡^ = 20.7 ± 1.1 mL/mol for the global apparent activation volumes of folding was obtained for DEN4-ED3 from the chevron plot (Figure 7D). This corresponds to about 40% of the value of ∆*V_f_*^0^ at equilibrium when referring to the same methyl resonance in 1D proton NMR steady-state experiments recorded under the same conditions (∆*V_f_*^0^ = 50.3 ± 2.6 mL/mol, Supplementary Materials, Figure S4). This result shows that less than half of the void volumes present in the folded state of DEN4-ED3 are formed already in the TSE, implying that the TSE is substantially hydrated. Besides, we obtained from the same chevron plot a value of *k*_f_^0^ = 1.14 (± 0.08) × 10^−3^ s^−1^ for the average kinetic constant of folding at ambient pressure. Note that, because amide and methyl groups probe different chemical environments, the apparent global value of ∆*V_f_*^0^ obtained from the unfolded-state methyl band differs significantly from the apparent residue-specific values of ∆*V_f_*^0^ measured from the native state amide resonances. 

## 3. Discussion

In this study, we monitored the pressure-induced unfolding of DEN4-ED3 with high-resolution NMR spectroscopy. From the analysis of the residue-specific denaturation curves, the first event in DEN4-ED3 unfolding was found to be the rupture of the junction between the N- and C-terminal ß-strands 1 and 9, on one edge of the ß-sandwich. Interestingly, this behavior is rather similar to what has been recently shown for the I27 domain of the multimodular giant protein Titin from the striated muscle cell, which also adopts an Ig-like fold, but with a significantly different sequence (sequence identity <7%) [27]. In Titin I27, the N- and C-termini are involved in ß-sheet structures: a small, distorted antiparallel (AB) ß-sheet, and a longer parallel (A’G) ß-sheet (where the capital letters stand for the ß-strands numbering, following the usual convention for Ig-like modules). The local pressure disruption of these sheets starts around 600 bar and concerns mainly these secondary structures at pressures up to 1000 bar. The local unfolding of these short ß-sheets has been proposed as the starting event of Titin Ig-like domain unfolding, yielding an intermediate folding state where the N-terminal strand is detached from the ß-sandwich. This partial unfolding of Ig-like modules of Titin in the I-band of the sarcomere has been proposed to contribute to the passive elasticity of striated muscle [30,31]. Beyond 1000 bar, the entire protein unfolds, due to water penetration into the hydrophobic core of the molecule. In the case of DEN4-ED3, the N- and C-terminal strands adopt a similar parallel orientation but do not form a parallel ß-sheet on one edge of the ß-sandwich, as for Titin I27. This might explain in part the difference in stability between the two proteins: addition of 1.7 M guanidine was necessary to decrease the stability of I27 sufficiently to ensure complete denaturation in the pressure range allowed by our experimental set-up (1–2500 bar), versus only 0.5 M in the case of DEN4-ED3. Despite the difference in stability, the starting event of unfolding remains strikingly similar for the two proteins: the disruption of the edge of the ß-sandwich bounded by the N- and C-terminal strands. As for Titin I27, the partial unfolding of DEN4-ED3 concerns only this region for a significant pressure range (600 to 1000 bar), suggesting that a reasonably stable intermediate state exists in the folding landscape of DEN4-ED3, where the N- and C-terminal strands are only loosely bound to the ß-sandwich. 

**Figure 8:**
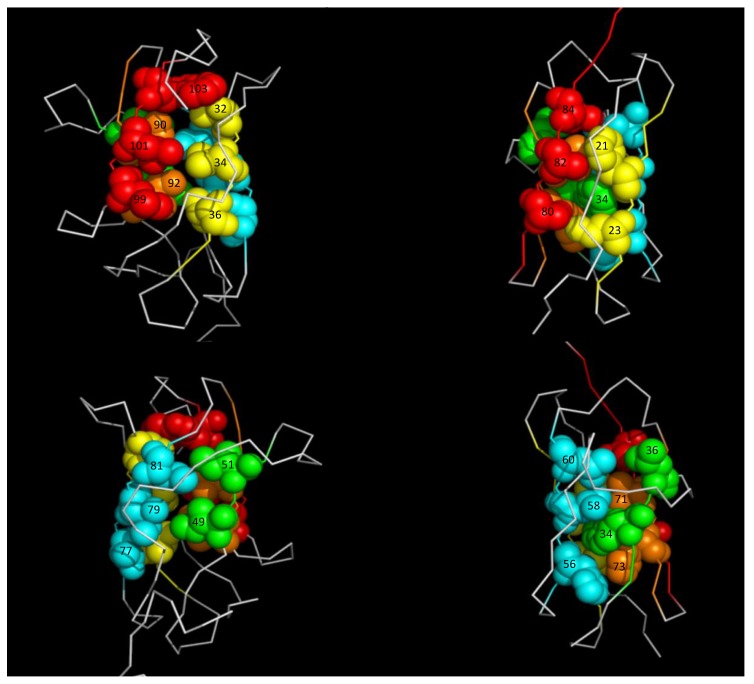
Comparison of the hydrophobic core of DEN4-ED3 and Titin I27. Two views related by a 180° rotation along the vertical axis of the hydrophobic core of (left) DEN4-ED3 and (right) Titin I27. The color coding is as follows: B (or ß2 for DEN4-ED3, according to Volk et al. [15]) strand, yellow; C (ß4) strand, green; E (ß7) strand, blue; F (ß8) strand, orange; G (ß9) strand, red. The labels stand for the residue numbers, as reported in the PDB (1TIU for Titin I27, 2H0P for DEN4-ED3).

Above 1000 bar, unfolding extends to the entire molecule, but in a different manner than for Titin I27. In the case of the I27 Ig-like module, the folded structure is completely destroyed above 1000 bar. In contrast, DEN4-ED3 unfolding progresses in a more stepwise manner, first affecting one sheet (ß1-ß2-ß7, or A-B-E, following the Ig-fold convention) of the ß-sandwich, with the structure of the second one (ß4-ß8-ß9, or C-F-G, following the Ig-fold convention) remaining preserved until a pressure of 1400 bar. This suggests that under hydrostatic pressure, water does not progress in a similar way within the hydrophobic core of the two proteins. DEN4-ED3 presents the characteristic core organization described by Clarke et al. [32] for Ig-like domains; the buried hydrophobic residues borne by strands 2 (B, in the usual Ig-fold convention) and 8 (F) are closely entangled and form the “spinal column” of the core, whereas hydrophobic residues from strands 4, 7, and 9 (C,E, and G) pack onto this central scaffold (Figure 8). Even if a similar hydrophobic core has been reported for Titin I27 [27], closer inspection reveals some differences: strand C contributes also to the spinal column of the hydrophobic core, with the indole ring of Trp34 being inserted between the buried hydrophobic residues from strands B and F. The remaining hydrophobic residues from strands E and G make a dense network of hydrophobic interactions around this compact core, mainly organized around Trp34 (Figure 8). It is tempting to speculate that the more concerted unfolding transition for Titin I27 is a consequence of the more integrated packing patterns of its core. Similarly, the difference in stability between the two proteins probably originates from the different organization of their hydrophobic core. Nevertheless, only extensive molecular dynamic simulations could bring precise insights on the side-chain reorganization in the core of the molecule under water progression.

This distinct core organization is likely responsible for the different hydration states of the TSEs for the two proteins as well. Indeed, kinetics experiments show that the TSE is considerably more hydrated, and hence less organized, in the case of DEN4-ED3 (∆*V*_f_^‡^ ≈ 40% of ∆*V_f_*^0^) than for Titin I27 (∆*V*_f_^‡^ ≈ 95% of ∆*V_f_*^0^). For both proteins, residue-specific values of activation volumes cannot be obtained, thus preventing definition of which part of the molecule is disordered and in interaction with solvent, and which part is ordered and dehydrated at the folding barrier. Nevertheless, based on ø value analysis, Fowler and Clarke [16] showed that the structure of I27 at the TSE was close to that of the folded state: only the A’ and G strands were completely unstructured, with ø values lower than 0.2, while all other strands of the ß sandwich were structured to some extent. This means that the structure of I27 at the TSE corresponds more or less to the structure of the intermediate state, as defined by HP-NMR analysis. Similarly, it is tempting to think that the 40% of dehydrated structures in the TSE might correspond to the ß4-ß8-ß9 (C-F-G) triple-stranded ß-sheet, the hydrated part consisting of the rest of the molecule, including the ß1-ß2-ß7 (A-B-E) sheet that unfolds at lower pressure.

Besides, our P-jump experiments yield a value of *k*_f_^0^ = 1.14 (± 0.08) × 10^−3^ s^−1^ for the rate constant for folding of DEN4-ED3, more than one order of magnitude lower than the value previously measured for Titin I27 (*k*_f_^0^ = 2.24 (± 0.11) × 10^−2^ s^−1^) [27]. This seems to corroborate the observation made by Clarke et al. [32], who found a positive linear correlation between the logarithm of the rate constant for folding and the stability of Ig-like protein.

## 4. Materials and Methods

### 4.1. Mutant Design, Expression, and Purification

The sequence of DEN4-ED3 was retrieved from UniProt (ID P09866, https://www.uniprot.org/) [33] and a synthetic gene encoding ED3′s sequence, a His6-tag and a thrombin cleavage site at the N-terminus, was cloned into a pET15b vector, as reported previously [13]. The ED3 domain was expressed in *Escherichia coli* BL21 (DE3) PLysS as inclusion bodies and purified as described [34,35]. In short, protein expression was induced by the addition of 1.0 mM isopropyl β-D- thiogalactopyranoside when the optical density at 590 nm reached 0.5. The cells were harvested by centrifugation and lysed by sonication. The ED3 domain contains two cysteines that were air-oxidized for 36 h at 37 °C in 6 M guanidine hydrochloride. Afterward, the His-tagged ED3 was purified by Ni-NTA (Qiagen, Venlo, The Netherlands) chromatography in the presence of 6 M guanidine hydrochloride, followed by overnight dialysis against 50 mM Tris-HCl (pH 7.0) at 4 °C. The His-tag was removed by thrombin (Sigma-Aldrich, St. Louis, MO, USA) cleavage (Supplementary Materials, Figure S5) and the protein was purified by a second passage through a Ni-NTA column followed by reverse-phase high-performance liquid chromatography (HPLC; Shimadzu Ltd., Tokyo, Japan). Uniform ^15^N labeling was obtained by growing cells in minimal M9 medium containing ^15^NH_4_Cl as the sole nitrogen source (Shoko Co. Ltd., Yokohama, Japan). Protein purity and identities were confirmed by analytical reversed-phase HPLC, and matrix-assisted laser desorption ionization time-of-flight mass spectroscopy (MALDI-TOF) using a TOF/TOF 5800 spectrometer (ABI SCIEX, Framingham, MA, USA) (Supplementary Materials, Figure S6). HPLC-purified proteins were lyophilized and stored at −30 °C.

### 4.2. Nuclear Magnetic Resonance

Samples for NMR measurements were prepared at about 0.5 mM (unlabeled protein for 1D NMR experiments) to 1 mM (^15^N-labeled protein for 2D heteronuclear NMR experiments) protein concentration in 10 mM Tris buffer (pH 7.0). The samples were centrifuged at 20,000× *g* for 5 min and at 4 °C to remove aggregates. The concentration and pH of the samples were confirmed just before performing the experiments, and 10% of D_2_O was added to the samples for deuterium lock. Except for H/D exchange experiments, 0.5 M of guanidinium chloride was added to all samples in order to permit unfolding of the protein within our accessible pressure range (1–2500 bar).

Unless otherwise specified in the text, all experiments were recorded at 10 °C on a Bruker AVANCE III 600 MHz (Wissembourg, France) equipped with a proton detection broad band inverse (BBI) 5 mm Z-gradient ^1^H-X probe head. Excitation Sculpting [36] and WATERGATE [37] (3D experiments) or coherence selection by gradient echoes (2D experiments) were used for water suppression. ^1^H chemical shifts were directly referenced to the methyl resonance of DSS (4,4-dimethyl-4-silapentane-1-sulfonic acid), while ^15^N chemical shifts were referenced indirectly to the absolute frequency ratio ^15^N/^1^H = 0.101329118. All NMR experiments were processed with Gifa [38].

### 4.3. Nuclear Magnetic Resonance Peak Assignment

Sequential assignment was obtained from the analysis of 3D {^1^H,^15^N} NOESY-HSQC (mixing time 100 ms) and TOCSY-HSQC (isotropic mixing: 60 ms) NMR double-resonance experiments recorded on uniformly ^15^N-labeled protein samples dissolved in 200 µL of buffer at a concentration of about 1 mM.

### 4.4. Proton/Deuteron Exchange Measurements

H/D exchange experiments were performed as previously described [39] with a 1 mM sample of ^15^N-labeled protein freshly dissolved in 200 µL D_2_O. A series of {^1^H-^15^N}-HSQC spectra were recorded at 600 MHz with a common measuring time of 15 min and a time limit of 96 hours. Amide proton protection factors [28] were calculated from the observed exchange rates (*k*_ex_) obtained from the time dependence of the peak intensities using an exponential decay.

### 4.5. High-Pressure Nuclar Magnetic Resonance Spectroscopy

Nuclear magnetic resonance spectra were acquired at 600 MHz using a 5 mm o.d. ceramic tube (330 µL of sample volume) from Daedelus Innovations (Aston, PA, USA). Hydrostatic pressure was applied to the sample directly within the magnet using the Xtreme Syringe Pump, also from Daedelus Innovations. 

### 4.6. Steady-State Studies

A series of 15 2D {^1^H-^15^N} HSQC based on coherence gradient selection were recorded at variable hydrostatic pressure (1, 50, 100, 300, 500, 700, 900, 1100, 1300, 1500, 1700, 1900, 2100, 2300, and 2500 bar), with a 2-h relaxation time after every pressure change, to allow the protein to reach full equilibrium. Relaxation times for the folding/unfolding reactions were previously estimated from series of 1D NMR experiments recorded after 200 bar P-jump, following the increase of the resonance band corresponding to the methyl groups in the unfolded state of the protein. The cross-peak intensities for the folded species were measured at each pressure, then fitted with a two-state model:

(1) $$I=\frac{I_{u}+I_{f}e^{-\left(\Delta G_{f}^{0}+p\Delta V_{f}^{0}\right)/RT}}{1+\;e^{-\left(\Delta G_{f}^{0}+p\Delta V_{f}^{0}\right)/RT}}$$

where *I* is the cross-peak intensity measured at a given pressure, and *I*_f_ and *I*_u_ correspond to the cross-peak intensities in the folded state (low pressure) and in the unfolded state (high pressure), respectively. $\Delta V_{f}^{0}$ and $\Delta G_{f}^{0}$ stand for the residue-specific apparent volume change at equilibrium and the free energy at atmospheric pressure, respectively. *I*_f_ and *I*_u_ values were floating parameters in the fit, and the data and fitted values were normalized (after the fit) using these plateau values to yield plots of the fraction folded as a function of pressure.

Native contact maps were obtained by using software CMView [40] with a threshold of 9 Å around the Cα of each residue, using the best structure among the 20 refined ones. Using the geometric mean, rather than the joint probability as done previously [18,21], ensures the correct unfolding profile in the case of two-state unfolding. 

### 4.7. Kinetics Studies

Global kinetic parameters were obtained through the analysis of series of 120 1D proton NMR spectra (32 scans each, 1 min duration) recorded as pseudo-2D experiments after positive 200 bar P-jumps. From the matrix obtained after Fourier transform of the 2D experiment along the acquisition dimension, the column at coordinate 0.96 ppm was extracted. The corresponding points describe the evolution with time of the intensity of the resonance belonging to unfolded methyl groups; they were fitted with a single-exponential growth model to extract the corresponding relaxation time *τ*. For a simple two-state reaction, this P-jump relaxation time *τ* corresponds to the inverse sum of the folding and unfolding rates (*τ*_(*p*)_ = 1/(*k*_u(*p*)_ + *k*_f(*p*)_)). The folding and unfolding rate constants (*k*_f_ and *k*_u_) are exponentially dependent on the pressure through the activation volume for the folding reaction (∆*V*^‡^_f_) and unfolding reaction (∆*V*^‡^_u_), respectively:

(2) $$k_{f(p)}=k_{f}e^{\frac{-p\Delta V_{u}^{‡}}{RT}}\;\mathrm{and}\;k_{u(p)}=k_{u}e^{\frac{-p\Delta V_{u}^{‡}}{RT}}$$

The pressure dependence of ln(*τ*_(*p*)_) was fitted using a nonlinear least-squares analysis to extract the values of the activation volume and folding/unfolding rates at atmospheric pressure, using the following equation for *τ*_(*p*)_:

(3) $$\tau \left(p\right)=\;{\left[k_{u}e^{\left(\frac{-p\Delta V_{u}^{‡}}{RT}\right)}+\;k_{u}K_{eq}e^{\left(\frac{-p\left(\Delta V_{f}^{0}+\Delta V_{u}^{‡}\right)}{RT}\right)}\right]}^{-1}$$

We constrained the ln(*τ*_(*p*)_) versus pressure fit using the previously determined equilibrium volume change $\Delta V_{f}^{0}$ and free energy for folding $\Delta G_{f}^{0}$ (see above) measured in the same conditions:

(4) $$\Delta G_{f}^{0}=\;-RTln\lceil \frac{k_{f}}{k_{u}}\rceil$$

with *K_eq_* = *k_f_*/*k_u_* and $\Delta V_{f}^{0}$ = $\Delta V_{f}^{‡}$ − $\Delta V_{u}^{‡}$.

## 5. Concluding Remarks

In this study, high-hydrostatic-pressure NMR was used to analyze at a residue-level resolution the folding pathway of DE4-ED3, an Ig-like protein domain from the dengue virus envelope. Interestingly, we found some similarity with the folding pathway of I27 module, another Ig-like domain found in Titin, a protein from the sarcomere of striated muscle cell. Notably, they share a common folding intermediate where the two N- and C-terminal strands are detached from the ß-sandwich. This finding represents an experimental confirmation of previous theoretical studies suggesting that folding pathways are conserved in protein families [41,42]. On the other hand, the kinetic studies performed on the two proteins show that, whereas the two proteins fold in a similar way, the structure of the Transition State Ensemble can be very different: dehydrated, with a structure close to the folded state in the case of Titin I27, or relatively hydrated, with an intermediary structure between the folded and unfolded state for DEN4-ED3.

In conclusion, it is of interest to see that two proteins with unrelated function that adopt a similar topology and a similar 3D fold (an Ig-like fold, in the present case) might have similar folding energy landscape, including similar folding intermediates, even though their sequences are completely different. This difference in primary structure might be responsible for the different folding kinetics observed for the two proteins, resulting in an earlier folding barrier for DEN4-ED3, and hence different structures at the Transition State Ensemble. 

## Supplementary Materials

The following are available online at https://www.mdpi.com/2218-273X/9/8/309/s1. Figure S1: Sequence alignment of the four serotypes of DEN-D3. Figure S2: Average amide chemical-shift variations. Figure S3: Representative examples of fits obtained for the decrease in intensity with pressure of HSQC cross-peaks. Figure S4: Steady-State experiments followed by High-Pressure 1D Proton NMR. Figure S5: SDS-PAGE analysis of {¹⁵N} His-DEN4 ED3 protein before and after thrombin cleavage. Figure S6: Analytical reverse phase HPLC chromatogram and MALDI-TOF spectrum of {¹⁵N} DEN4 ED3. Table S1: Sequential and structural similarity among ED3’s of four dengue serotypes. Table S2: Exchange rates and Protection Factors (PF) measured for amide protons involved in the main chain hydrogen bonds stabilizing the ß-sheets found in DEN4-ED3.
