# Supplementary material for: Folding of the Ig-Like Domain of the Dengue Virus Envelope Protein Analyzed by High-Hydrostatic-Pressure NMR at a Residue-Level Resolution
Source: Biomolecules. 2019 Jul 26;9(8):309. doi: 10.3390/biom9080309 (PMC6723665; doi:10.3390/biom9080309)
Supplement: Supplementary file 1 [file biomolecules-09-00309-s001.pdf]

biomolecules

*Supplementary materials*

**Table S1.** (A) Exchange rates and Protection Factors (PF) measured for amide protons involved in the main chain hydrogen bonds stabilizing the ß-sheets found in DEN4-ED3 (column 1). The numbers given in column 2 and 3 stand for the residue numbers of residues bearing the HN amide groups (donor) and the C=O carbonyl group (acceptor) involved in the hydrogen bond, respectively.

| ß-sheet | Donor (HN) | Acceptor (C=0) | $k_{ex}$ ($min^{-1}$) | +/- | PF | +/- | log(PF) | +/- |
|---|---|---|---|---|---|---|---|---|
| ß1 - ß2 | **19** | 37 | 0.00024015 | 0.000025111 | 281000 | 26600 | 5.4487 | 0.094662 |
| | **22** | 44 | | | | | | |
| | **25** | 33 | | | | | | |
| | **27** | 31 | | | | | | |
| | **33** | 25 | 0.021539 | 0.0020828 | 369 | 32.5 | 2.567 | 0.088076 |
| | **37** | 19 | 0.060076 | 0.013766 | 276 | 51.5 | 2.4409 | 0.18659 |
| | **44** | 22 | | | | | | |
| ß2 - ß7 | **32** | 81 | | | | | | |
| | **34** | 79 | 0.000014149 | 0.000036761 | 256000 | 185000 | 5.4082 | 0.72266 |
| | **36** | 77 | 0.0002122 | 0.000025128 | 31100 | 3290 | 4.4928 | 0.10579 |
| | **77** | 36 | 0.040561 | 0.0057809 | 382 | 47.6 | 2.5821 | 0.12461 |
| | **79** | 34 | 0.0078069 | 0.00036378 | 1250 | 55.7 | 3.0969 | 0.04456 |
| | **81** | 32 | 0.00016631 | 0.000049409 | 28100 | 6440 | 4.4487 | 0.22918 |
| ß7 - ß5 | **62** | 82 | | | | | | |
| | **64** | 80 | | | | | | |
| | **65** | 80 | | | | | | |
| | **82** | 62 | | | | | | |
| ß3 - ß6 | **45** | 70 | 0.030191 | 0.0034243 | 1700 | 170 | 3.2304 | 0.1 |
| | **47** | 68 | 0.0096568 | 0.00071391 | 684 | 47.1 | 2.8351 | 0.068859 |
| | **70** | 45 | | | | | | |
| ß4 - ß8 | **50** | 91 | | | | | | |
| | **52** | 89 | 0.0018881 | 0.000135 | 9410 | 628 | 3.9736 | 0.066738 |
| | **89** | 52 | 0.00024186 | 0.000036277 | 41300 | 5390 | 4.616 | 0.13051 |
| | **91** | 50 | 0.000016164 | 0.00017437 | 182000 | 167000 | 5.2601 | 0.91758 |
| | **93** | 48 | 0.0005641 | 0.00008017 | 48800 | 6070 | 4.6884 | 0.12439 |
| ß8 - ß9 | **88** | 103 | 0.00086799 | 0.000051457 | 44800 | 2510 | 4.6513 | 0.056027 |
| | **90** | 101 | | | | | | |
| | **92** | 99 | 0.000010643 | 0.000082627 | 318000 | 282000 | 5.5024 | 0.88679 |
| | **99** | 92 | | | | | | |
| | **101** | 90 | 0.0016711 | 0.000080169 | 6260 | 287 | 3.7966 | 0.045847 |
| | **103** | 88 | 0.015803 | 0.001807 | 4180 | 429 | 3.6212 | 0.10263 |

**Table 2.** Sequential and structural similarity among ED3's of four dengue serotypes. [1].

| Percent identity/ Cα RMSD (Å) | DEN1-ED3 | DEN2-ED3 | DEN3-ED3 | DEN4-ED3 |
|---|---|---|---|---|
| DEN1-ED3 (PDB: 3IRC) | | 0.87 Å | 0.57 Å | 1.01 Å |
| DEN2-ED3 (PDB: 3UZV) | 79% | | 0.74 Å | 0.76 Å |
| DEN3-ED3 (PDB: 3VTT) | 88% | 78 % | | 1.07 Å |
| DEN4-ED3 (PDB: 3WE1) | 71% | 77 % | 70 % | |

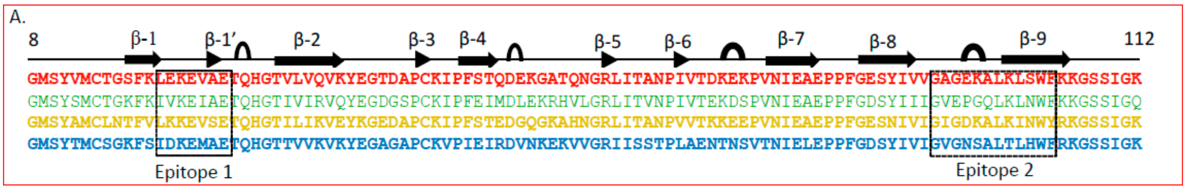


**Figure S1.** (A) Sequence alignment of four serotypes of DEN ED3. (DEN1-ED3: red, DEN2-ED3: green DEN3-ED3: yellow, DEN4-ED3: blue). The residues are numbered according to Volk et al, 2007 [2] (BMRB code 7087). Both the sequences and structures were retrieved from the PDB-IDs given in the table. The sequences start at Gly 8 and ends at Lys 112 (corresponding to Gly 294 and Lys 398, respectively, in Elahi et al. [3]). The secondary structures indicated on the top are calculated using 3WE1 and DSSP (http://swift.cmbi.ru.nl/gv/dssp/). The epitope regions are those shown in Lisova et al. [1].

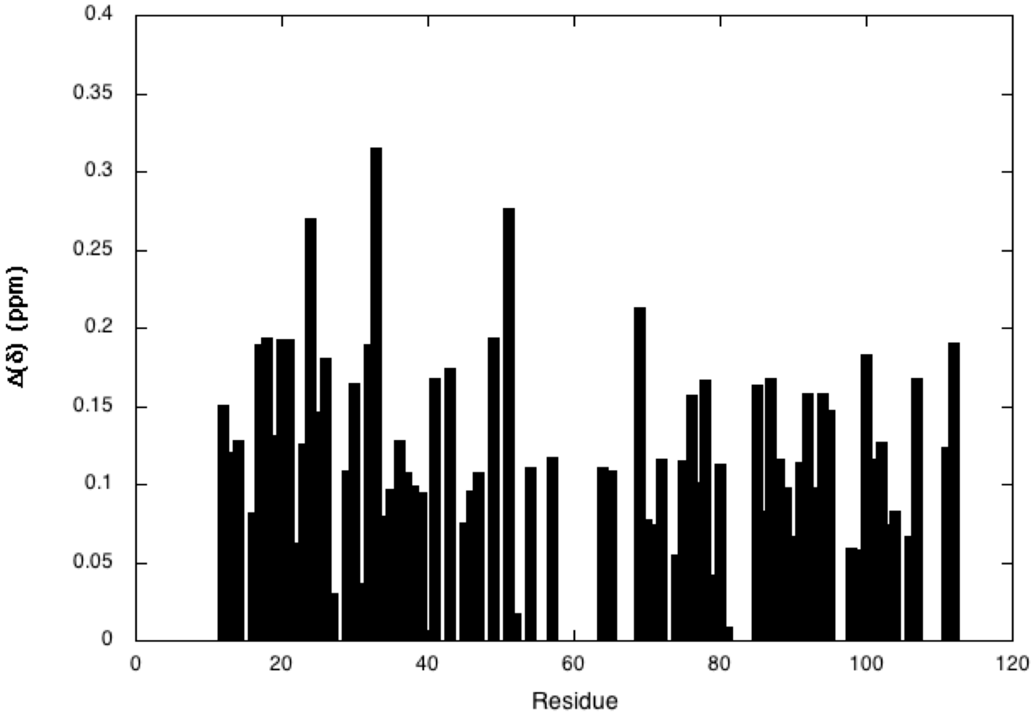


**Figure S2.** (A) (Right) Average amide chemical-shift variations, calculated as $\Delta\delta = \sqrt{\Delta\delta_H{}^2 + \left(\frac{\Delta\delta_N}{5}\right)^2}$, measured between spectra recorded under the experimental conditions used in the present

denaturation study and under the conditions used for the previously published NMR structural study (25°C, 50mM Tris pH 7.5, 50 mM NaCl) ([1]; BMRB code 7087).

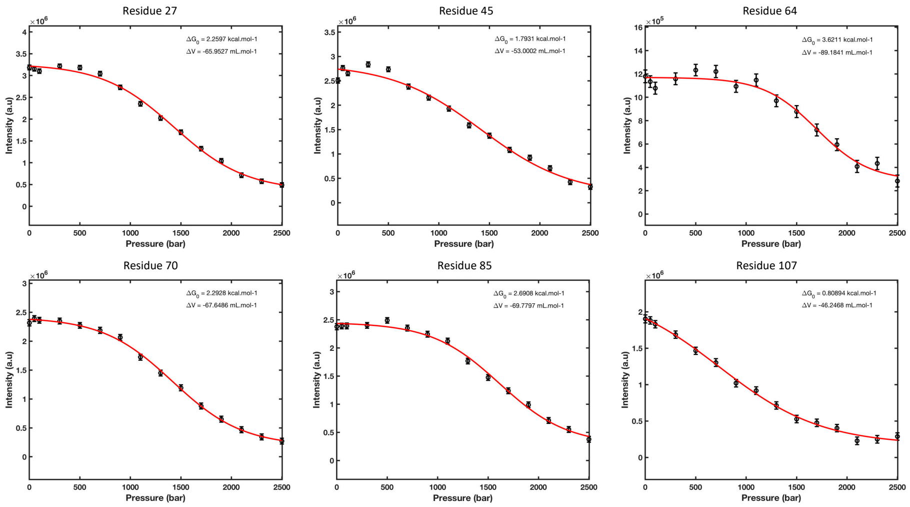


**Figure S3.** Six representative examples (-90 < $\Delta V_u^0$ < -40 ml/Mol) of fits obtained for the decrease in intensity with pressure of cross-peaks from the corresponding residues (indicated on top of each graph) with Equation 1 (Materials and Methods) implemented on a in-house MATLAB software.

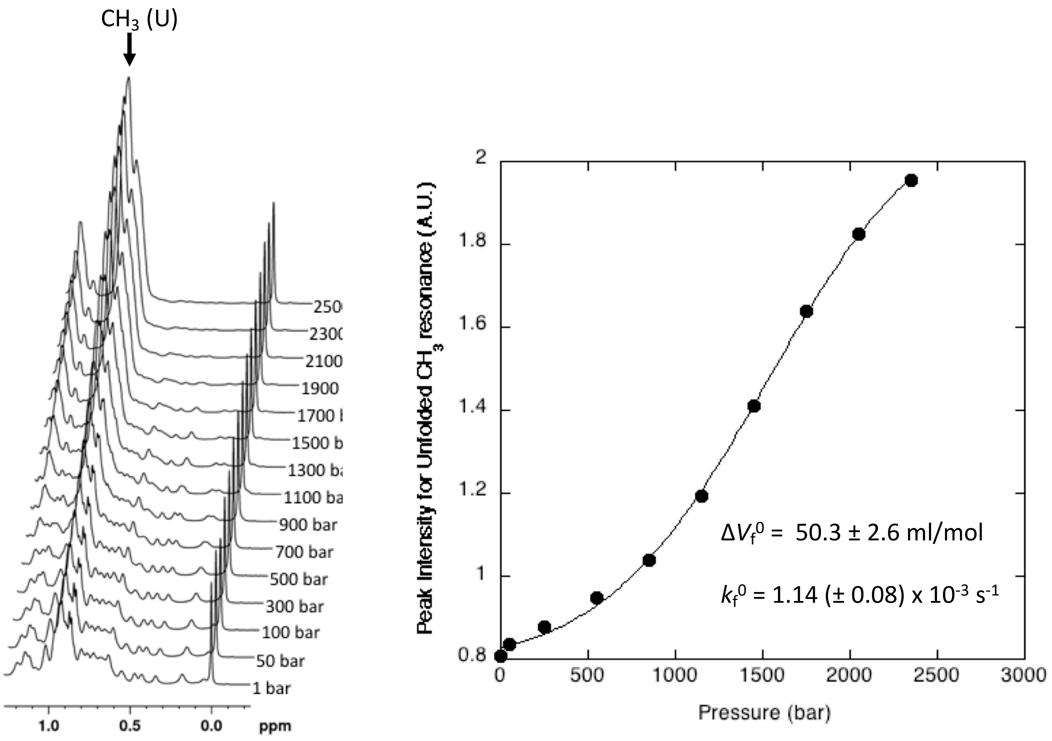


**Figure S4.** Steady-State experiments followed by High-Pressure 1D Proton NMR. (Left) Stacked plot of the 1D proton NMR spectra recorded on DEN4-ED3 at increasing pressure. The 1D NMR

experiments are recorded once the steady-state is reached, 2 hours after the *P*-Jump. Only the region of the methyl resonances is reported. The arrow indicates the resonance corresponding to unfolded methyl groups ($CH_3$ (U)), used as probe to monitor the folding/unfolding equilibrium. (Right) Denaturation curves obtained for DEN4-ED3. The curves were fitted with Equation 1 to obtain the values of $\Delta V_f^0$ and $\Delta G^0$ reported in the insert.

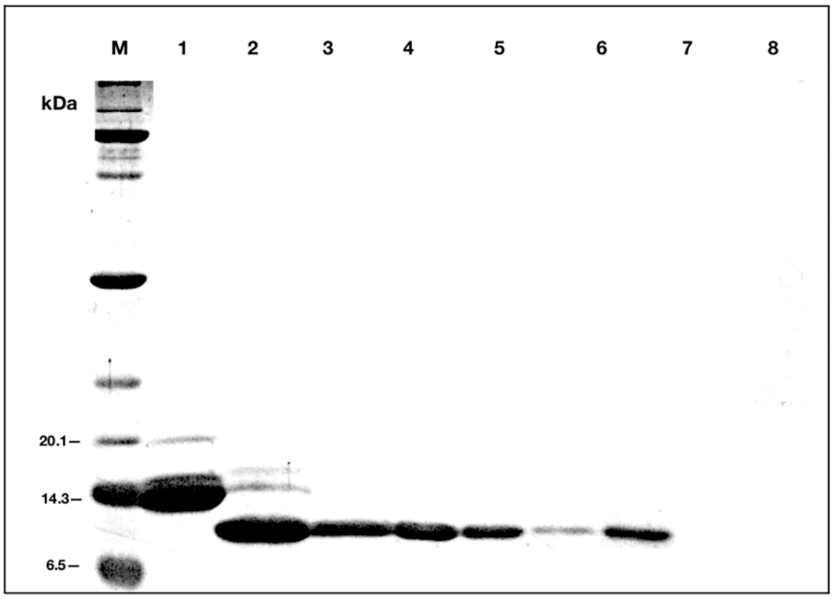


**Figure S5.** SDS-PAGE analysis of [15N] His-DEN4 ED3 protein before and after thrombin cleavage. **M** represents the molecular weight marker (protein marker, low molecular weight range, FUJIFILM Wako chemicals). Lane-**1**: [15N] His-DEN4 ED3 protein; Lane-**2**: His-tag cleaved [15N] DEN4 ED3 protein; Lanes **3-8** show the His tag purification of ED3 after thrombin cleavage of the His tag. Lanes **3-5** and Lanes **6-8** indicate, respectively, the wash and the elution fractions.

**A.**

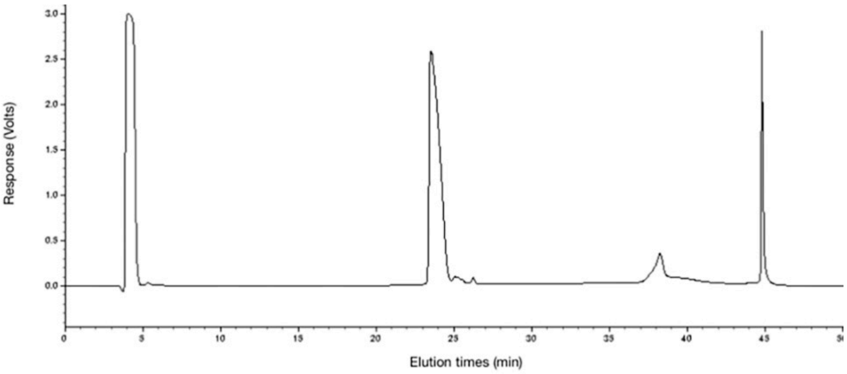

**B.**

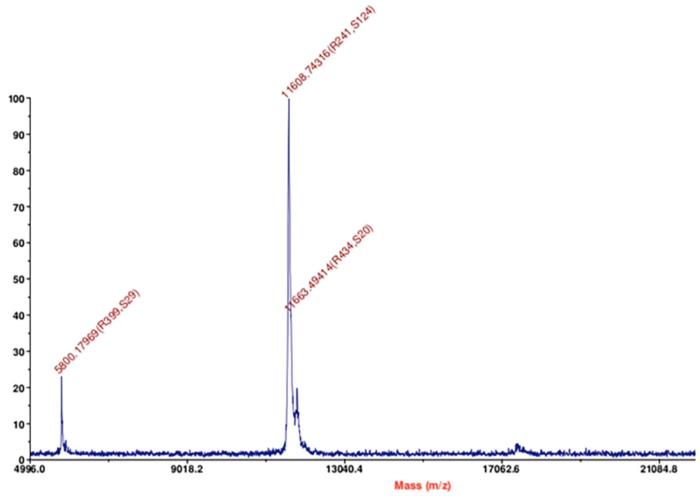


**Figure S6.** (**A**) Analytical reverse phase HPLC chromatogram of [$^{15}$N] DEN4 ED3 (**B**) MALDI-TOF spectrum of [$^{15}$N] DEN4 ED3. The molecular mass was 11608.74 Dalton (Calculated molecular mass=11603.00 Dalton).

## References


1. Lisova, O.; Hardy F.; Petit, V.; Bedouelle, H. Mapping to completeness and transplantation of a group-specific, discontinous, neutralizing epitope in the envelope protein of dengue virus. *J. Gen. Virol.* **2007**, *88*, 2387-2397.
2. Volk, D.E.; Lee, Y.C.; Li, X.; Thiviyanathan, V.; Gromowski, G.D.; Li, L.; Lamb, A.R.; Beasley, D.W.C.; Barrett, A.D.T.; Gorenstein, D. G Solution structure of the envelope protein domain III of dengue-4 virus. *Virology* **2007**, *364*, 147–154.
3. Elahi, M.; Islam, M.M.; Noguchi, K.; Yohda, M.; Toh, H.; Kuroda. Y. Computational prediction and experimental characterization of a "size switch type repacking" during the evolution of dengue envelope protein domain III (ED3). *Biochim. Biophys.* **2014**, *1844*, 585–592.


© 2019 by the authors. Submitted for possible open access publication under the terms and conditions of the Creative Commons Attribution (CC BY) license (http://creativecommons.org/licenses/by/4.0/).
